# The health economic–industrial complex: production and innovation for universal health access, Brazil

**DOI:** 10.2471/BLT.23.290838

**Published:** 2024-04-17

**Authors:** Carlos AG Gadelha, Gabriela Maretto, Marco AC Nascimento, Felipe Kamia

**Affiliations:** a Secretary of Science, Technology, Innovation and Health Economic-Industrial Complex, Ministry of Health, Esplanada dos Ministérios, Bloco G, 70058-900, Brasília, DF, Brazil.; b Fiocruz’s Center for Strategic Studies, Rio de Janeiro, Brazil.

## Abstract

**Problem:**

The coronavirus disease 2019 (COVID-19) pandemic has highlighted global disparities in accessing essential health products, demonstrating the critical need for low- and middle-income countries to develop local production and innovation capabilities.

**Approach:**

The health economic–industrial complex approach changed the values that guided innovation and industrial policies in Brazil. The approach directed health production and innovation to universal access; the health ministry led a whole-of-government approach; and public procurement was strategically applied to stimulate productive public and private investments. The institutional, technological and productive capacities built up by the health economic–industrial complex allowed the country to quickly establish local COVID-19 vaccines production and guarantee access for the population.

**Local setting:**

Brazil has a universal health system that guarantees access to health for its 215 million population.

**Relevant changes:**

Public policies and actions, based on the health economic–industrial complex, guided investment projects in line with health demands, strengthened local producers, and increased autonomy in the production of health products in areas of greater technological dependence. During the COVID-19 pandemic, the country was able to rapidly scale up local vaccine production. By August 2021, Brazil had produced 74.8% (151 463 502/202 437 516) of the vaccine doses used in the country.

**Lessons learnt:**

The Brazilian example shows that low- and middle-income countries can build systemic development policies that increase their capability to produce and innovate in concert with universal health systems. This increased capacity can guarantee access to health products and supplies that are critical during global health emergencies.

## Résumé

**Problème:**

La pandémie de maladie à coronavirus 2019 (COVID-19) a mis en lumière les disparités mondiales en matière d'accès aux produits médicaux essentiels, soulignant la nécessité, pour les pays à revenu faible et intermédiaire, de développer des capacités locales de production et d'innovation.

**Approche:**

Le complexe économique-industriel de la santé a redéfini les valeurs qui guident les politiques d'innovation et les politiques industrielles au Brésil. Ce complexe a réorienté la production et l'innovation dans le domaine de la santé vers un accès universel; le Ministère de la Santé a adopté une approche pangouvernementale; et enfin, les marchés publics ont été passés de manière stratégique afin de stimuler les investissements productifs dans les secteurs public et privé. Les capacités institutionnelles, technologiques et productives développées par le complexe économique-industriel de la santé ont permis au pays d'établir rapidement une production locale de vaccins contre la COVID-19 et d'en assurer l'accès à la population.

**Environnement local:**

Le Brésil possède un système de santé universel permettant à ses 215 millions d'habitants de bénéficier d'une prise en charge médicale.

**Changements significatifs:**

Les politiques et actions publiques fondées sur le complexe économique-industriel de la santé ont donné lieu à des projets d'investissement correspondant aux exigences sanitaires, soutenu les producteurs locaux et favorisé une autonomie accrue dans la fabrication de produits médicaux au sein des régions plus dépendantes sur le plan technologique. Durant la pandémie de COVID-19, le pays a pu rapidement accroître sa production de vaccins. En août 2021, le Brésil produisait 74,8% (151 463 502/202 437 516) des doses utilisées sur son territoire.

**Leçons tirées:**

L'exemple du Brésil montre que les pays à revenu faible et intermédiaire peuvent instaurer des politiques de développement systémiques qui renforcent leurs capacités de production et d'innovation, et vont de pair avec des systèmes de santé universels. Cette augmentation des capacités est susceptible de garantir un accès aux équipements et produits médicaux essentiels en cas d'urgence sanitaire mondiale.

## Resumen

**Situación:**

La pandemia de la enfermedad por coronavirus de 2019 (COVID-19) ha evidenciado las disparidades mundiales en el acceso a productos sanitarios esenciales, demostrando la necesidad crítica de que los países de ingresos bajos y medios desarrollen capacidades locales de producción e innovación.

**Enfoque:**

El enfoque del complejo económico e industrial de la salud cambió los valores que guiaban la innovación y las políticas industriales en Brasil. El enfoque orientó la producción y la innovación sanitarias hacia el acceso universal; el ministerio de salud lideró un enfoque de todo el gobierno; y la contratación pública se aplicó estratégicamente para estimular las inversiones productivas públicas y privadas. Las capacidades institucionales, tecnológicas y productivas creadas por el complejo económico e industrial de la salud permitieron al país establecer rápidamente la producción local de vacunas contra la COVID-19 y garantizar el acceso de la población.

**Marco regional:**

Brasil tiene un sistema sanitario universal que garantiza el acceso a la salud a sus 215 millones de habitantes.

**Cambios importantes:**

Las políticas y acciones públicas, basadas en el complejo económico e industrial de la salud, orientaron los proyectos de inversión en función de las demandas sanitarias, reforzaron a los productores locales y aumentaron la autonomía en la producción de productos sanitarios en las áreas de mayor dependencia tecnológica. Durante la pandemia de la COVID-19, el país fue capaz de escalar rápidamente la producción local de vacunas. En agosto de 2021, Brasil había producido el 74,8% (151 463 502/202 437 516) de las dosis de vacunas utilizadas en el país.

**Lecciones aprendidas:**

El ejemplo brasileño demuestra que los países de ingresos bajos y medios pueden crear políticas de desarrollo sistémicas que aumenten su capacidad de producir e innovar en colaboración con los sistemas sanitarios universales. Esta mayor capacidad puede garantizar el acceso a productos y suministros sanitarios que son fundamentales durante las emergencias sanitarias mundiales.

## ملخص

**المشكلة:**

أدت جائحة مرض فيروس كورونا 2019 (كوفيد 19) إلى تسليط الضوء على التفاوتات العالمية في الحصول على الخدمات الصحية الأساسية، مما يشير إلى الحاجة الماسة لدى الدول ذات الدخل المنخفض والدخل المتوسط لتطوير قدرات الإنتاج والابتكار المحلية.

**الأسلوب:**

أدى أسلوب المجمع الاقتصادي الصناعي الصحي إلى تغيير القيم التي ارتكزت عليها سياسات الابتكار والسياسات الصناعية في البرازيل. وقد قام هذا الأسلوب بتوجيه الإنتاج والابتكار في مجال الخدمات الصحية لتصبح متاحة للحصول عليها بشكل موسع؛ وقادت وزارة الصحة أسلوبًا يعتمد على جميع المؤسسات الحكومية؛ وتم تطبيق المشتريات العامة بشكل استراتيجي لتحفيز الاستثمارات المنتجة العامة والخاصة. إن القدرات المؤسسية والتكنولوجية والإنتاجية، التي نتجت عن المجمع الصناعي والاقتصادي الصحي، سمحت للدولة بتأسيس إنتاج محلي للقاحات كوفيد 19 بسرعة، مع ضمان حصول السكان على هذا الإنتاج.

**المواقع المحلية:**

تتمتع البرازيل بنظام صحي شامل يضمن حصول سكانها، البالغ عددهم 215 مليون نسمة، على الخدمات الصحية.

**التغيّرات ذات الصلة:**

قامت السياسات والإجراءات العامة، المعتمدة على المجمع الاقتصادي الصناعي الصحي، بتوجيه المشروعات الاستثمارية بما يتماشى مع المتطلبات الصحية، ودعم المنتجين المحليين، وزيادة الاستقلالية في تقديم الخدمات الصحية في المناطق ذات الاعتماد الأكبر على التكنولوجيا. خلال جائحة كوفيد 19، تمكنت الدولة من توسيع إنتاج اللقاحات المحلية بسرعة. بحلول أغسطس/آب 2021، أنتجت البرازيل %74.8 (151463502/202437516) من جرعات اللقاح المستخدمة في الدولة.

**الدروس المستفادة:**

يوضح هذا المثال البرازيلي أن الدول ذات الدخل المنخفض والدخل المتوسط بإمكانها بناء سياسات تنموية نظامية مما يزيد قدرتها على الإنتاج والابتكار بالتنسيق مع النظم الصحية الشاملة. ويمكن لهذه القدرة المتزايدة أن تضمن الحصول على الخدمات والإمدادات الصحية شديدة الأهمية خلال حالات الطوارئ الصحية العالمية.

## 摘要

**问题:**

新型冠状病毒肺炎 (COVID-19) 疫情突出了全球在获得基本健康产品方面的差异性问题,让我们看到,中低收入国家迫切需要发展当地生产和创新能力。

**方法:**

健康经济工业综合体这一方法改变了指导巴西制定创新和工业政策所秉持的价值观。该方法旨在确保全民均可因健康生产和创新而受益;卫生部牵头实施政府政策;战略性地进行公共采购以刺激生产性公共和私人投资。健康经济工业综合体的制度、技术和生产能力确保该国能够迅速在当地建立新型冠状病毒肺炎疫苗生产基地,并保证人们都能获得疫苗。

**当地状况:**

巴西的全民健康系统可确保其 2.15 亿民众都能获得健康服务。

**相关变化:**

巴西的公共政策和措施,以健康经济工业综合体为基础,根据健康需求指导开展投资项目,提升了当地生产商的能力,并在技术依赖程度较高的地区促进了自主生产健康产品。在新型冠状病毒肺炎疫情期间,该国能够迅速扩大当地疫苗生产。截至 2021 年 8 月,巴西的疫苗生产量达到了全国疫苗使用量的 74.8% (151,463,502/202,437,516)。

**经验教训:**

巴西的例子表明,中低收入国家可以制定系统的发展政策,提高与全民健康系统相协调的生产和创新能力。通过提高这些能力,可以确保在全球出现健康突发事件的时候能够获得至关重要的健康产品和用品。

## Резюме

**Проблема:**

Пандемия коронавирусной инфекции 2019 года (COVID-19) выявила глобальные различия в доступе к основным медицинским товарам, продемонстрировав критическую необходимость для стран с низким и средним уровнем дохода в развитии местного производства и инновационного потенциала.

**Подход:**

Подход к экономике здравоохранения и промышленному комплексу изменил ценности, которыми руководствуются специалисты по инновациям и промышленной политике в Бразилии. Этот подход направляет производство и инновации в сфере здравоохранения на обеспечение всеобщего доступа, Министерство здравоохранения возглавляет общегосударственный подход, а государственные закупки стратегически используются для стимулирования продуктивных государственных и частных инвестиций. Институциональный, технологический и производственный потенциал, созданный в рамках экономики здравоохранения и промышленного комплекса, позволил стране быстро наладить местное производство вакцины COVID-19 и обеспечить доступ населения к ней.

**Местные условия:**

В Бразилии действует универсальная система здравоохранения, которая гарантирует доступ к медицинским услугам 215 миллионам жителей страны.

**Осуществленные перемены:**

Государственная политика и действия, основанные на экономике здравоохранения и промышленном комплексе, направили инвестиционные проекты в соответствии с потребностями здравоохранения, укрепили местных производителей и повысили автономию в производстве товаров для здоровья в областях с большей технологической зависимостью. Во время пандемии COVID-19 страна смогла быстро расширить масштабы местного производства вакцин. К августу 2021 года в Бразилии было произведено 74,8% (151 463 502/202 437 516) доз вакцины, используемых в стране.

**Выводы:**

На примере Бразилии видно, что страны с низким и средним уровнем дохода могут выстраивать системную политику развития, которая повышает их способность производить и внедрять инновации в сочетании с универсальной системой здравоохранения. Расширение возможностей может гарантировать доступ к медицинским товарам и материалам, которые крайне важны во время чрезвычайных ситуаций в области здравоохранения.

## Introduction

During the coronavirus disease 2019 (COVID-19) pandemic, global disparities in production and innovation capacities resulted in the lack of access to essential health products and services, such as COVID-19 vaccines, revealing and reinforcing global health inequities.^1^ Despite the global initiatives created to tackle these disparities, national interests hindered effective international cooperation.^2^ This fallout underscored the importance of local production and innovation capacities for essential health products and services in low- and middle-income countries, especially during global health emergencies.^3^ Hence, governments in low- and middle-income countries need to create policies that stimulate local production and innovation to meet the health needs of their populations.^4^

Since 2008, the Brazilian government has developed public policies for health products and services based on the health economic–industrial complex, an approach that integrates production of health products, innovation and access to health care. The adoption of this approach encompassed institutional and policy changes aimed at strengthening the state's capacity to coordinate and implement industrial and science, technology and innovation policies. These policies have boosted local production and innovation capacities towards the health needs of the population.^5^

This article outlines how, over the past decades, public policies based on the health economic–industrial complex approach enabled the Brazilian government to quickly establish local production of COVID-19 vaccines, thereby ensuring access for the population.

## Local setting

Brazil, a large upper-middle-income country, has a universal health system that guarantees access to health care for its 215 million population.^6^ Established in the 1988 Constitution of Brazil, the health system is based on the principle of health as a citizen's right and a duty of the state. The national immunization programme, created in 1973 and strengthened with the creation of the universal health system, has led to high vaccination coverage, especially considering the country’s large territory.^7^ A key aspect in the success of the programme has been its ability to link vaccine accessibility with the development of national production capacities of vaccines.^8^

## Approach

Access to health is conditioned by access to the goods and services needed to fulfil the citizen's right to health. Therefore, the expansion of the health system without a national strategy for health production and innovation tends to reinforce countries' economic and technological dependence, further hindering access to health.^1^^,^^5^

In the context of this conclusion, the health economic–industrial complex approach, developed in the early 2000s in the *Fundação Oswaldo Cruz* (Fiocruz), probed how universal access to health could be achieved in a middle-income country with a welfare state still in formation.^5^ Initially, the work focused on vulnerabilities within the universal health system, particularly the increasing trade deficits on health goods linked to the expansion of access to health care. The demand for these health goods served as a foundation to build an economy for health for all. This work depended on a complex political economy that integrates the epidemiological setting, health system organization, strengthening of national capacities within science, technology, innovation and production, as well as geopolitics and international trade in health.^1^^,^^5^

By adapting the concept to practices in the real world, the theory of the complex evolved into a solid public policy approach. In 2008, the health economic–industrial complex was incorporated as a reference to guide the formulation of comprehensive and articulated industrial and science, technology and innovation policies of the health ministry. Up to 2015, several policies stemming from the health economic–industrial complex were introduced, such as the List of Strategic Products, which signalled the needs of the universal health system to the multiple actors and institutions involved in the process of innovation and production in health. This list was periodically defined by the Executive Group of the health economic-industrial complex, which was an inter-ministerial governance group led by the health ministry, with the participation of public and private entities and civil society. The executive group became the locus for the institutional coordination of health economic–industrial complex policies, linking with the different actors and institutions related to innovation and production in health, including regulatory and financing institutions. Therefore, the executive group legitimized the construction of a whole-of-government approach guided by national challenges and social missions.^9^^,^^10^

Another key initiative was the strategic use of the state's purchasing power, to subordinate industrial and science, technology and innovation policies to meet the demands of the universal health system.^5^ The most consistent and innovative examples of the complex’s actions are the public–private partnerships for domestic production of health goods to meet health needs. The health ministry guarantees that partnerships gain access to the public market, provided there is a transfer of technology for the product in question. The market share and duration of access, which ranges from 5 to 10 years, depends on the technological complexity of the product being transferred to the local public partner institutions from the private sector. The products eligible for the partnerships were determined by the List of Strategic Products.

## Relevant changes

The public policies and actions, based on the health economic–industrial complex, guided investment projects in line with health demands, strengthening local producers, public and private, and increasing autonomy in the production of health products in areas of greater technological dependence.^10^ The accumulation of technological capabilities of public producers was fundamental for the institutions to rapidly incorporate COVID-19 vaccine production technologies in a global context of dispute over asymmetric vaccine distribution.^11^

**Fig. 1:**
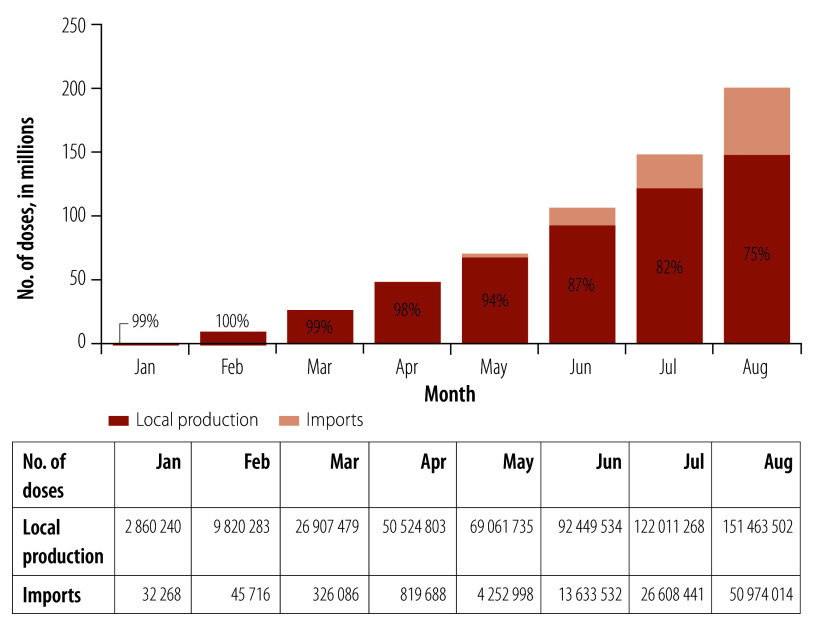
Vaccination rollout by vaccine origin, Brazil, 2021

**Fig. 2:**
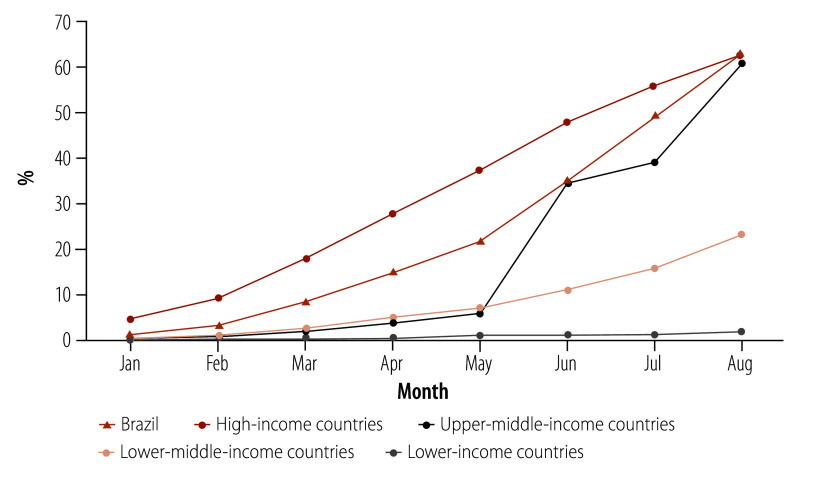
Share of population with at least one dose of COVID-19 vaccine, 2021

The institutional, technological and productive capacities built up by previous health economic–industrial complex public policies, enabled the rapid scale-up of local production capacity of COVID-19 vaccines. Even in the context of the asymmetric global vaccine distribution, by the end of August 2021, Brazil reached a slightly higher coverage of at least one dose of COVID-19 vaccine (63.2%) than the average for high-income countries (62.8%). This achievement can be attributed to the local production of 74.8% (151 463 502/202 437 516) of the vaccine doses (Fig. 1). At that time, only 1.7% of the population in low-income countries, 23.1% in lower-middle-income countries and 61.0% in upper-middle-income countries had received at least one dose of the vaccine (Fig. 2). 

Data source: Brazilian health ministry.^12^

COVID-19: coronavirus disease 2019.

Data source: Mathieu et al.^13^

## Lessons learnt

The Brazilian example demonstrates that low- and middle-income countries can create systemic development policies that increase their capacity to produce and innovate health products and services aligned with the needs of a universal health system. To achieve this, governments must broaden the health policy scope to coordinate innovation and production strategies in the health sector. This broadness can be applied in a local, regional and global context (Box 1).

Box 1 **Summary of main lessons learnt**

- The existence of production, innovation and institutional capacities is essential to ensure universal access to health and to tackle global health challenges
- Broadening the scope of health policies will help to coordinate innovation and production strategies in health and develop emergency preparedness
- The health economic–industrial complex is a concrete example of how to develop industrial and science, technology and innovation policies that promote universal access to health care

Overcoming the political and institutional division between socio-environmental, industrial and science, technology and innovation and economic policies is imperative to achieve health for all. The policies created based on the health economic–industrial complex approach serve as important examples of public policy that integrated economic, science, innovation and technology dimensions with social needs. The COVID-19 response highlighted the link between institutional, scientific and productive capabilities with the capacity to ensure access to health products and services amid global competition.

The fourth industrial revolution, marked by new technologies in digitalization, automation and connectivity, along with climate change^14^ and rising social inequalities, is transforming social needs and introducing new challenges.^10^ Therefore, public policies and international cooperation should pursue an integrated sustainable development vision that includes the ecological and digital transition of health systems. Industrial and science, technology and innovation policies are essential and structural for developing preparedness for health emergencies, including those associated with climate change.

The main challenges regarding the implementation of public policies based on the health economic–industrial complex approach are maintaining institutional stability to manage technological risks; protecting investments in health production and innovation; and ensuring an industrial and science, technology and innovation strategy able to link innovation with production capabilities. 

The recent launch of the National Strategy for the health economic–industrial complex in Brazil, within a mission-oriented industrial and innovation policy framework, updates and strengthens this approach. The national strategy incorporates lessons learnt during this public policy process and aligns national efforts with global health challenges, including those efforts associated with the digital and ecological transition. Now more than ever, effective international cooperation is essential to overcome asymmetries that constrain the global health agenda. Brazil can contribute to raise the urgency of integrating innovation, production and health access in the global health agenda by sharing the possibilities of this concrete public policy experience in global and regional forums. 

The COVID-19 pandemic has made it clear that an agenda integrating health production, innovation and access is vital for preparedness against current and future public health challenges. To improve autonomy and ensure universal access to health, the Brazilian government has included the health economic–industrial complex as a strategic axis in Brazil's technological and industrial development policy. Additionally, the World Health Organization Council on the Economics of Health for All recognized this complex as a successful example of a mission-oriented policy to guarantee health for all.^9^
